# Case Report: Fulminant sintilimab-associated myocarditis complicated by complete atrioventricular block and suspected myositis-myasthenia gravis overlap syndrome

**DOI:** 10.3389/fcvm.2026.1968961

**Published:** 2026-10-29

**Authors:** Pian Cheng, Xiaolin Zhao, Min Jin, Qinghua Lai, Jie Liu

**Affiliations:** 1 School of Clinical Medicine, Shandong Second Medical University, Weifang, China; 2 Department of Respiratory Medicine, Central Hospital Affiliated to Shandong First Medical University, Jinan, China; 3 Department of Oncology, Central Hospital Affiliated to Shandong First Medical University, Jinan, China

**Keywords:** cardiac troponin T, complete atrioventricular block, immune checkpoint inhibitor-associated myocarditis, myositis–myasthenia gravis overlap syndrome, sintilimab

## Abstract

Immune checkpoint inhibitor (ICI)-associated myocarditis can cause life-threatening conduction disturbances and may coexist with neuromuscular immune-related adverse events. We report a 67-year-old man with stage IIIA lung squamous cell carcinoma who developed fatigue, bilateral ptosis, myalgia, weakness, and palpitations 13 days after his first dose of sintilimab following concurrent chemoradiotherapy. On admission, cardiac troponin T (cTnT) was 874.2 ng/L and creatine kinase-MB exceeded 300 μg/L. Serial electrocardiograms showed progression from bundle branch and fascicular block to complete atrioventricular block, profound bradycardia, and shock, despite a left ventricular ejection fraction of 54%. Coronary angiography revealed no obstructive coronary disease. The findings supported a clinical diagnosis of sintilimab-associated myocarditis with clinically suspected myositis–myasthenia gravis overlap syndrome; cardiac magnetic resonance imaging and confirmatory neuromuscular investigations were not performed. Treatment included intravenous methylprednisolone, immunoglobulin, mycophenolate mofetil, temporary cardiac pacing, and two limited-volume plasma exchange sessions (600 mL each). Infliximab (300 mg per infusion) was subsequently administered empirically for persistent myocardial biomarker elevation. Sinus rhythm returned, and cardiopulmonary and neuromuscular symptoms improved. Following withdrawal of immunosuppression, chest discomfort recurred alongside a cTnT rise to 299.4 ng/L, prompting resumption of corticosteroid-based therapy. At the latest follow-up on April 30, 2026, the patient remained asymptomatic with stable cardiac function, although cTnT remained mildly elevated. In ICI-associated myocarditis, a preserved ejection fraction does not exclude life-threatening conduction disease, and myocardial injury may recur after immunosuppression is withdrawn; continued ECG and troponin surveillance is therefore warranted.

## 1 Introduction

Immune checkpoint inhibitors (ICIs) have substantially reshaped the treatment landscape of non-small cell lung cancer (NSCLC) (1). However, immune-related adverse events (irAEs), particularly cardiovascular toxicities, remain an important clinical concern. ICI-associated myocarditis is uncommon, with a reported incidence of 0.06%–3.8%, but carries a high mortality rate of approximately 39.7%–66% (2, 3). The incidence after PD-1 inhibitor monotherapy is approximately 0.5% and may be higher with combination regimens (2–6). Myocarditis may coexist with myositis and myasthenia gravis, forming a potentially fatal overlap syndrome (6, 7). Outcomes may be particularly poor in older patients, with mortality reported to reach 83.3% in those aged ≥65 years (8). In this report, we describe an elderly patient who developed fulminant sintilimab-associated myocarditis complicated by clinically suspected immune-mediated myocarditis–myositis–myasthenia gravis overlap syndrome (IM3OS) and complete atrioventricular block. We further detail the longitudinal clinical course, emphasizing the challenges of managing severe conduction disturbances and mitigating recurrent myocardial injury during the tapering of immunosuppressive therapy.

## 2 Case presentation

A 67-year-old man presented with blood-streaked sputum on December 12, 2024, and was diagnosed with stage IIIA (T4N0M0) squamous cell carcinoma of the right lung on December 20, 2024. His medical history included more than 10 years of hypertension and sinus arrhythmia and an 8-month history of right-sided facial paralysis. He received concurrent chemoradiotherapy from December 25, 2024, to February 6, 2025, consisting of thoracic radiotherapy (60 Gy in 30 fractions) and weekly paclitaxel 100 mg for five doses (Table 1). Radiotherapy dosimetry showed a mean heart dose of 10.96 Gy and a heart V30 of 9.12%. After a 29-day interval following radiotherapy, sintilimab 200 mg was administered on March 7, 2025, as the first cycle of maintenance immunotherapy. Before the first sintilimab infusion on March 7, 2025, ECG showed sinus rhythm, left-axis deviation, and T-wave abnormalities (Figure 1A). Cardiac troponin T was 7.84 ng/L (reference range, 0–14 ng/L), and BNP was 23.85 pg/mL. Baseline total creatine kinase, NT-proBNP, and transthoracic echocardiography, including GLS assessment, were not obtained.

**Table 1:** Timeline of chemotherapy and immunotherapy.

Time Point	Event
2024-12-25 to 2025-02-06	Radiotherapy period
2024-12-27 to 2025-01-28	Chemotherapy period
2025-02-06 to 2025-03-06	Washout period: 29 days
2025-03-07	Initiation of sintilimab

**Figure 1:**
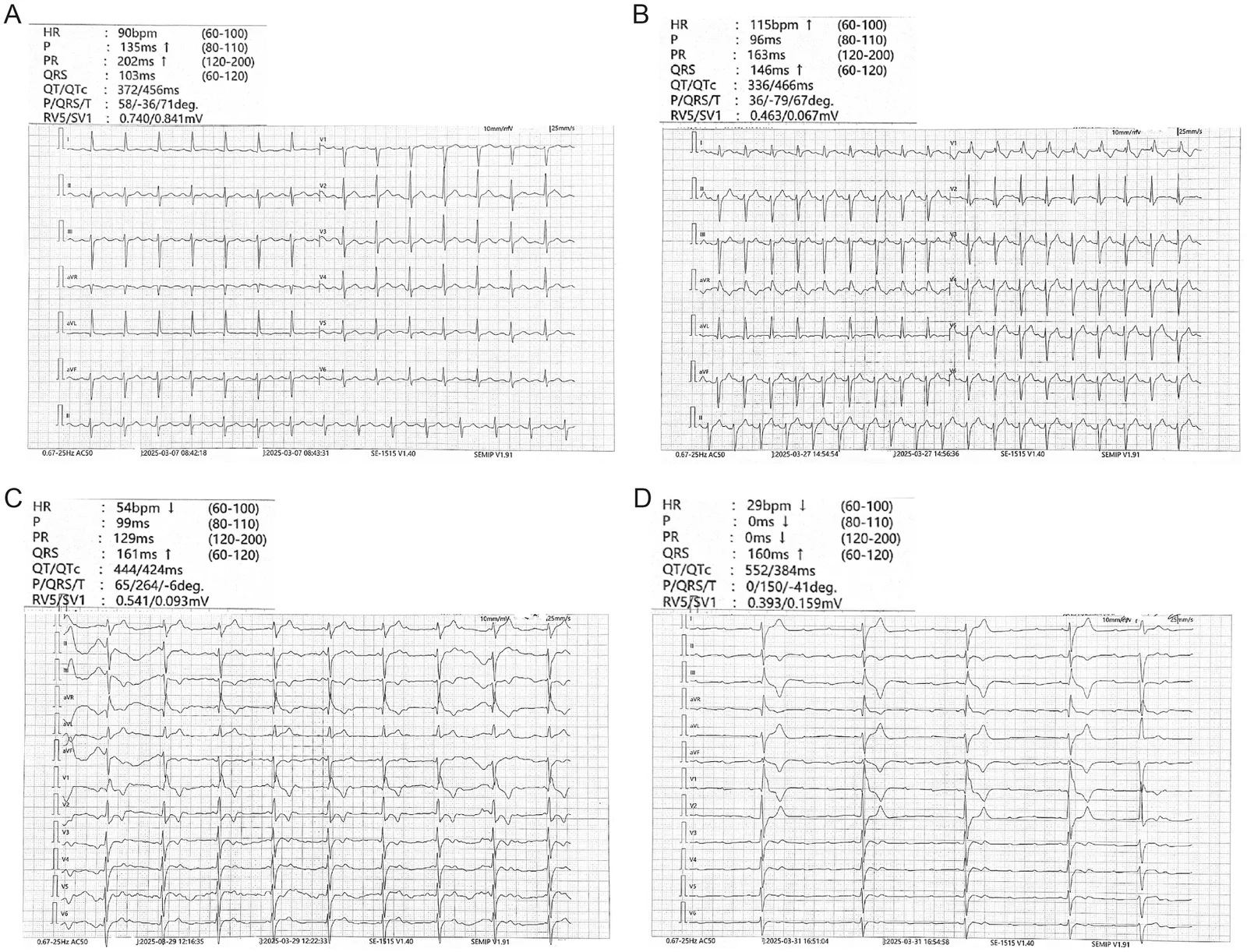
Serial electrocardiographic changes demonstrating progressive conduction abnormalities following sintilimab administration. **(A)** Baseline electrocardiogram (ECG) on March 7, 2025, showing sinus rhythm, left-axis deviation, and T-wave abnormalities. **(B)** ECG on March 27, 2025, showing sinus tachycardia, complete right bundle branch block (RBBB), and left anterior fascicular block. **(C)** ECG on March 29, 2025, showing complete atrioventricular block with an escape rhythm and complete RBBB. **(D)** ECG on March 31, 2025, showing persistent complete atrioventricular block with profound bradycardia (approximately 29 beats/min).

Thirteen days after sintilimab administration, the patient developed fatigue, anorexia, bilateral ptosis, blurred vision, myalgia, limb weakness, and intermittent palpitations without fever or other viral prodromal symptoms. He was admitted on March 27. His temperature was 36.7 °C, heart rate 82 beats/min, blood pressure 159/87 mmHg, height 1.68 m, and weight 75 kg. Bilateral ptosis was evident, and muscle strength was grade 4/5 in all four limbs. Laboratory testing showed cTnT 874.2 ng/L, creatine kinase-MB (CK-MB) > 300 μg/L (reference range, 0–4.87 μg/L), alanine aminotransferase 335 U/L, and aspartate aminotransferase 979.7 U/L. Creatinine was 69 μmol/L, and serum sodium, potassium, chloride, calcium, and magnesium were within reference ranges. Thyroid-stimulating hormone and free thyroxine were within reference ranges. Plasma cortisol was 262.8 nmol/L at 08:00 and 537.5 nmol/L at 24:00, while adrenocorticotropic hormone was <0.22 pmol/L at both time points. Nucleic acid tests for coxsackievirus, adenovirus, influenza virus, parainfluenza virus, Epstein–Barr virus, and SARS-CoV-2 were negative. Pulmonary function testing and objective respiratory muscle assessment were not performed.

ECG on March 27 demonstrated sinus tachycardia, complete right bundle branch block, and left anterior fascicular block (Figure 1B). Transthoracic echocardiography showed interventricular septal thickening, regional left ventricular wall-motion abnormalities, and a left ventricular ejection fraction (LVEF) of 54%. Assessment of bulbar involvement was complicated by pre-existing right-sided facial paralysis with chronic dysarthria. The patient did not report dysphagia or chewing weakness. Electromyography, neostigmine testing, pulmonary function testing, and objective respiratory muscle assessment were not performed because of clinical deterioration and the patient's reluctance to undergo additional investigations in the context of substantial treatment-related expenses. Acetylcholine receptor antibody testing was unavailable at our institution. Cardiac magnetic resonance imaging and endomyocardial or muscle biopsy were not performed during the acute phase because of clinical instability. Based on the temporal relationship to ICI exposure, marked myocardial injury, progressive conduction abnormalities, and associated neuromuscular symptoms, sintilimab-associated myocarditis with clinically suspected myositis–myasthenia gravis overlap syndrome was considered.

Intravenous methylprednisolone 300 mg/day was initiated promptly. On March 29, the patient developed marked bradycardia (30–40 beats/min), chest tightness, chest pain, dyspnea, and dizziness. Respiratory muscle weakness could not be established because the dyspnea could also be explained by the severe cardiac involvement. ECG demonstrated complete atrioventricular block with a junctional escape rhythm and complete right bundle branch block (Figure 1C). At this time, cTnT had increased to 971.1 ng/L, whereas CK-MB had decreased to 113.6 μg/L. Electrolyte testing showed sodium 132 mmol/L, potassium 3.8 mmol/L, chloride 104 mmol/L, calcium 1.02 mmol/L, and magnesium 0.90 mmol/L. He was transferred to the intensive care unit, where methylprednisolone was increased to 500 mg/day for 3 days, intravenous immunoglobulin 30 g/day was administered for 5 days, and mycophenolate mofetil was started at 2 g/day. Coronary angiography on March 30 showed no obstructive coronary stenosis and TIMI grade 3 flow in the major epicardial vessels, making acute obstructive coronary syndrome unlikely.

Temporary pacemaker implantation was initially recommended but declined by the family. On March 31, chest symptoms and dyspnea worsened, blood pressure fell to 88/56 mmHg, and profound bradycardia persisted with complete atrioventricular block (Figure 1D). Repeat electrolyte testing did not reveal a major disturbance sufficient to explain the conduction abnormality. A temporary pacemaker was subsequently implanted. Given the unstable cardiovascular condition, two limited-volume plasma exchange procedures were performed on April 1 and 2, each replacing 600 mL of plasma with fresh frozen plasma, corresponding to approximately 20% of the estimated plasma volume per session. Sinus rhythm recovered by April 5, and the temporary pacemaker was removed on April 8.

Despite improvement in symptoms and cardiac rhythm, myocardial injury markers remained elevated. After transfer back to the oncology department, tumor necrosis factor-α was 14.32 pg/mL (reference <8 pg/mL). In the setting of persistent myocardial injury despite corticosteroids, intravenous immunoglobulin, mycophenolate mofetil, and limited-volume plasma exchange, immunosuppression was further escalated with infliximab 300 mg per infusion (approximately 4 mg/kg) on April 10, April 25, and May 27. Corticosteroids were gradually tapered, mycophenolate mofetil was continued, and trimethoprim–sulfamethoxazole (TMP-SMX) 0.5 g/day was administered for Pneumocystis jirovecii prophylaxis. By May 12, cTnT had decreased to 154.9 ng/L and CK-MB to 6.86 μg/L.

Two microbiologically documented episodes of pneumonia occurred during prolonged immunosuppression. The first occurred on April 16 while the patient was receiving methylprednisolone 80 mg/day; Klebsiella pneumoniae was isolated, and meropenem 1 g every 8 h was administered until May 4. The second occurred on June 10 while he was receiving oral methylprednisolone 40 mg/day; Klebsiella pneumoniae and Candida albicans were identified, and meropenem 1 g every 8 h plus fluconazole 0.2 g/day was continued until June 22. No Pneumocystis jirovecii infection was detected. cTnT declined to 90.72 ng/L by June 30. Methylprednisolone, mycophenolate mofetil, and TMP-SMX were discontinued on July 21 after gradual tapering.

On August 28, the patient was readmitted with recurrent chest discomfort. Laboratory testing on August 29 showed cTnT 299.4 ng/L and CK-MB 6.77 μg/L. Intravenous methylprednisolone 80 mg/day was restarted together with oral mycophenolate mofetil and TMP-SMX. Echocardiography showed no major structural abnormality and an LVEF of 61%. Follow-up chest CT showed marked regression of the right hilar lesion compared with pre-immunotherapy imaging (Figure 2). Cardiac biomarkers again declined, and methylprednisolone was transitioned to an oral taper; cTnT was 128 ng/L on September 12. At the latest follow-up on April 30, 2026, the patient remained free of recurrent cardiopulmonary or neuromuscular symptoms, with stable cardiac function, although cTnT remained mildly elevated at approximately 50–60 ng/L. The overall treatment course, cardiac events, immunosuppressive therapy, infectious complications, and biomarker trajectory are summarized in Figure 2C and Figure 3.

**Figure 2:**
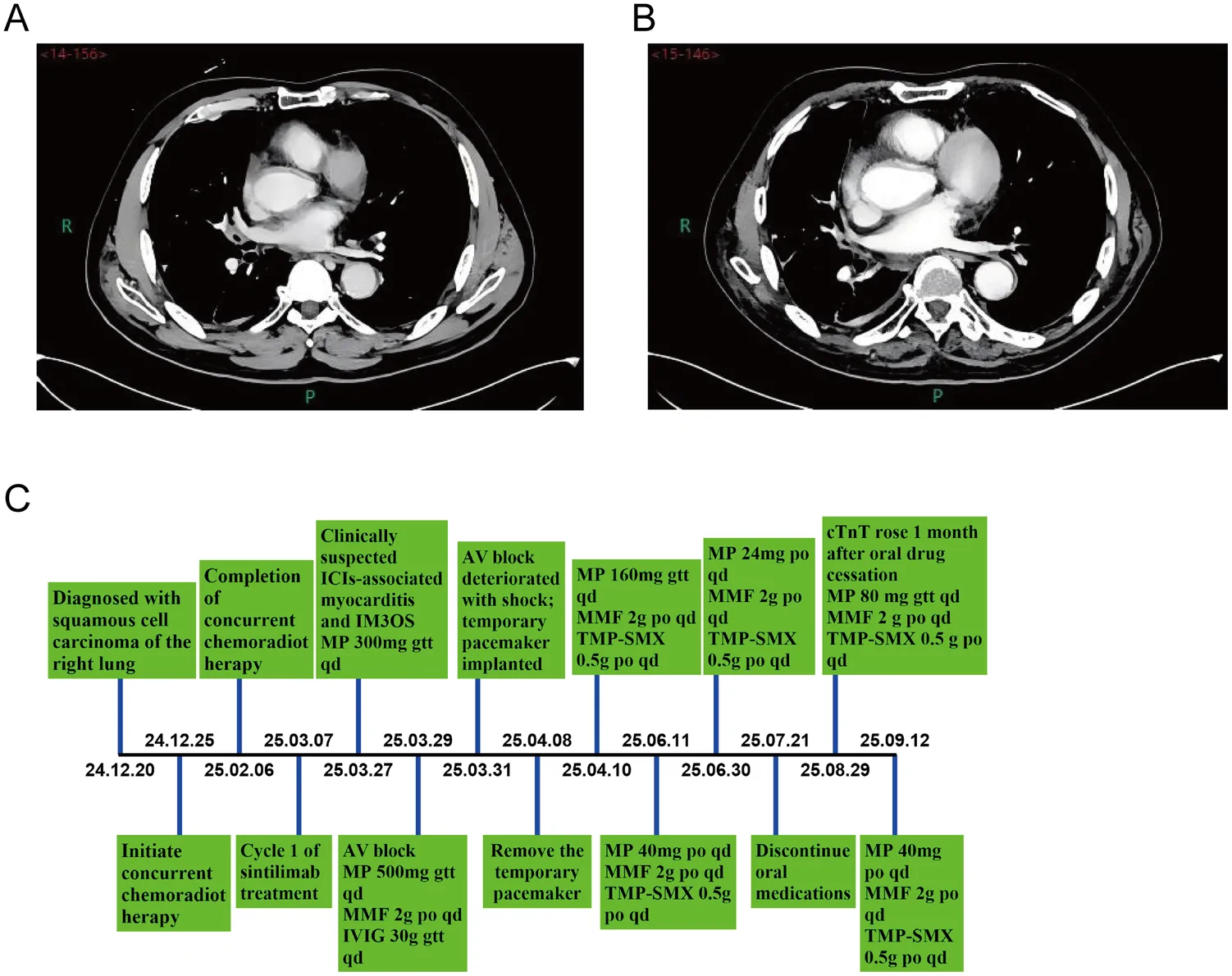
Tumor response imaging and clinical treatment timeline. **(A)** Baseline chest contrast-enhanced CT (March 6, 2025) obtained after concurrent chemoradiotherapy and before immunotherapy initiation, showing a nodular low-density lesion in the right pulmonary hilum (cross-sectional diameter: 19 mm × 9 mm). **(B)** Follow-up chest contrast-enhanced CT (August 29, 2025) after immunotherapy, demonstrating marked regression of the hilar lesion with only localized linear opacities, confirming a complete response (CR). **(C)** Timeline summarizing key diagnostic events and clinical interventions. MP, methylprednisolone; MMF, mycophenolate mofetil; IVIG, intravenous immunoglobulin.

**Figure 3:**
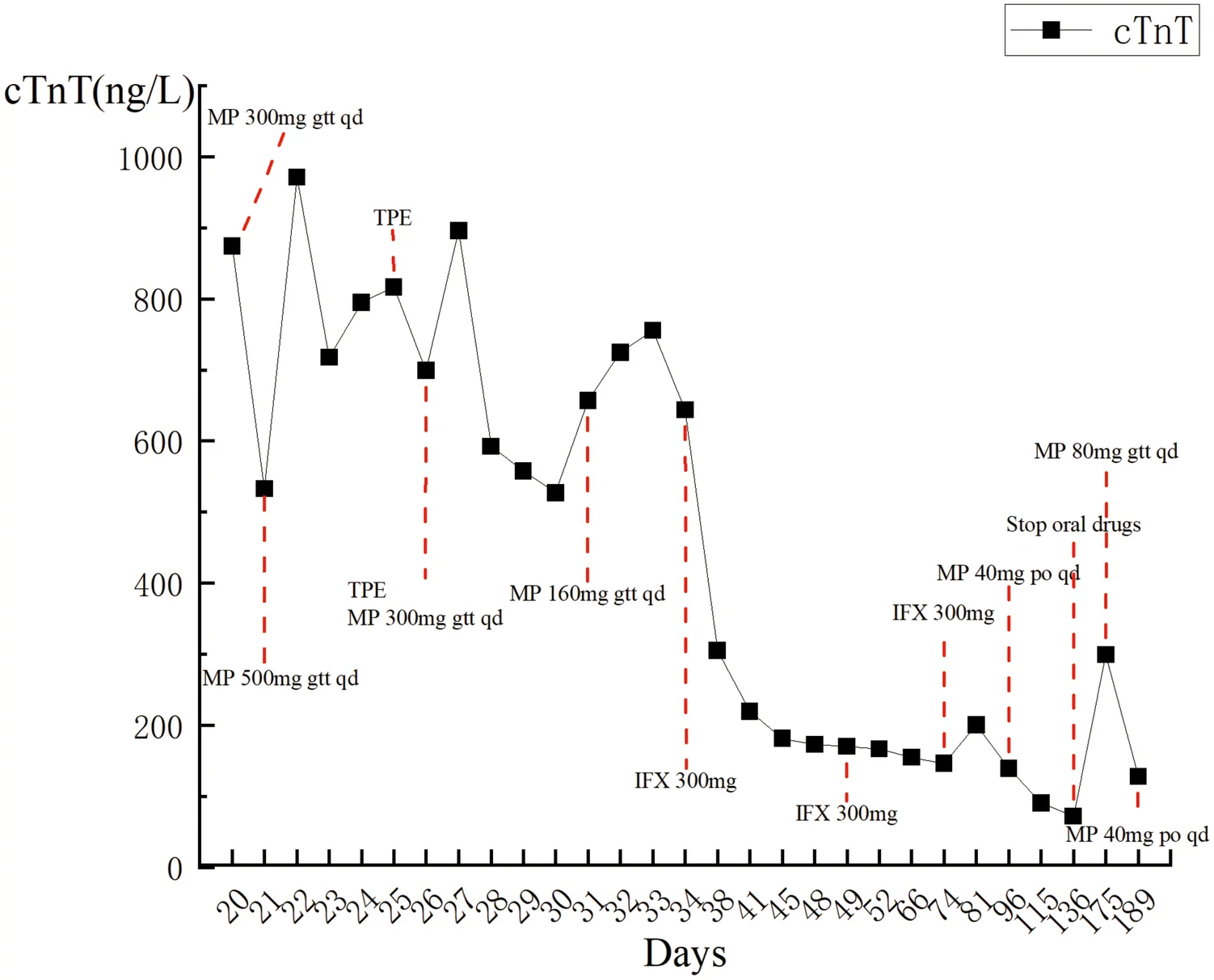
Serial cardiac troponin T (cTnT) levels and corresponding interventions. Serum cTnT (ng/L) trend over days (*x*-axis: days since immunotherapy initiation; black squares). Key interventions are indicated by red dashed lines: methylprednisolone (MP) therapy, therapeutic plasma exchange (TPE), and infliximab (IFX). On day 31 after immunotherapy, the intravenous steroid dose was tapered by 25%–30% every 5–10 days, reaching 30 mg by day 63. This intravenous regimen was maintained until day 96, when oral steroid therapy was initiated. The MP dose was reduced by 8 mg every 7 days from day 96 to day 136, and discontinued on day 136. After 189 days, the MP dose was further reduced by 8 mg every 7 days, and maintained at 16 mg.

Interpretation of possible bulbar involvement was complicated by pre-existing right-sided facial paralysis with chronic speech articulation difficulties. The patient did not report dysphagia or chewing weakness.

## 3 Discussion

ICI-associated myocarditis is an uncommon but potentially fatal cardiovascular immune-related adverse event, and its diagnosis is particularly challenging when cardiac manifestations coexist with skeletal-muscle and neuromuscular symptoms. In the present case, the close temporal relationship between sintilimab exposure and the onset of fatigue, ptosis, myalgia, weakness, palpitations, marked myocardial biomarker elevation, new conduction abnormalities, and ventricular wall-motion abnormalities strongly supported an immune-mediated process. Several alternative causes of myocardial injury were considered. Coronary angiography demonstrated no obstructive epicardial coronary disease, making acute coronary syndrome unlikely (9). Viral nucleic acid testing was negative for several common cardiotropic and respiratory viruses (10), while electrolyte abnormalities sufficient to explain the progressive conduction disturbance were not identified. Thyroid function tests did not identify an overt thyroid disorder that could explain the presentation (11). Echocardiography did not demonstrate the characteristic pattern of Takotsubo cardiomyopathy. Prior thoracic radiotherapy and paclitaxel exposure nevertheless represent important potential contributors to cardiovascular vulnerability. Radiation-related cardiac injury is usually delayed and is influenced by cardiac dose exposure (12–15), whereas paclitaxel may be associated with arrhythmias and ventricular dysfunction (16–18). In this patient, the mean heart dose was 10.96 Gy and V30 was 9.12%; however, the possibility of interaction between prior chemoradiotherapy and subsequent ICI exposure cannot be completely excluded, particularly because the cardiovascular effects of combined thoracic radiotherapy and immunotherapy remain incompletely defined (19). The pre-sintilimab cTnT level was within the reference range, although hypertension, pre-existing rhythm abnormalities, baseline ECG changes, and the absence of a pre-chemoradiotherapy echocardiogram limit assessment of pre-existing subclinical cardiac injury (20–22).

The coexistence of ptosis, myalgia, and generalized weakness raised concern for concomitant neuromuscular immune-related adverse events (23). Approximately 1% of ICI-treated patients may develop myocarditis, and available evidence suggests that approximately 25% of patients with ICI-associated myocarditis have concomitant myositis and 11% have concomitant myasthenia gravis (7, 24, 25). These percentages do not represent the incidence of the complete three-component overlap syndrome. Patients presenting with suspected ICI-associated myocarditis should therefore undergo targeted assessment for concurrent myositis and myasthenia gravis, including CK measurement, relevant autoantibody testing, electrophysiological studies, and respiratory muscle evaluation when clinically indicated and feasible.

According to the diagnostic framework proposed by Bonaca et al. (26), the documented clinical syndrome, new cardiac troponin elevation, and dynamic ECG abnormalities support at least possible myocarditis. Although regional wall-motion abnormalities were identified, the absence of baseline echocardiography prevented confirmation that these abnormalities were new. CMR and myocardial biopsy were unavailable, limiting further diagnostic classification. Similarly, the absence of confirmatory neuromuscular investigations prevented a definitive diagnosis of IM3OS. Accordingly, the overlap syndrome remained clinically suspected.

Conduction-system involvement progressed rapidly. The ECG, which showed sinus rhythm with left-axis deviation and T-wave changes before sintilimab, demonstrated bifascicular block on admission and complete atrioventricular block 2 days later, followed by profound bradycardia and hemodynamic collapse. Conduction disturbances are a recognized manifestation of ICI-associated myocarditis, and atrioventricular block is among the more serious rhythm complications reported with PD-1 inhibitor therapy (27–30). Electrolytes were normal or near-normal and the coronary arteries were patent, arguing against a metabolic or ischemic cause. Despite high-dose methylprednisolone, the patient progressed to severe bradycardia and shock and needed temporary pacing; sinus rhythm returned several days later and the pacemaker was removed. Notably, his LVEF at presentation was 54% — a reminder that near-normal systolic function offers no protection against life-threatening electrical instability in this disease. Conduction abnormalities in suspected ICI myocarditis therefore warrant serial ECG monitoring and early cardiology involvement, regardless of ejection fraction.

Corticosteroids remain the cornerstone of treatment for ICI-associated myocarditis. In this patient, intravenous methylprednisolone was initially administered empirically at 300 mg/day before the development of complete atrioventricular block and hemodynamic shock. This dose was below the 500–1,000 mg/day regimen recommended by the 2022 ESC cardio-oncology guidelines for suspected ICI-associated myocarditis. Following rapid clinical deterioration, methylprednisolone was increased to 500 mg/day for three days, and intravenous immunoglobulin and mycophenolate mofetil were added (31–34). Because myocardial injury markers remained elevated, two limited-volume plasma exchange procedures were subsequently performed, each exchanging 600 mL of plasma, substantially less than a conventional therapeutic plasma exchange volume. Given the patient's hemodynamic instability, this represented an individualized intervention, although its independent therapeutic contribution cannot be determined (33, 34). Infliximab was subsequently introduced empirically at 300 mg per infusion (approximately 4 mg/kg) because myocardial injury markers remained elevated despite multimodal treatment. An elevated TNF-α level was also documented, but it was measured after treatment initiation and clinical stabilization and therefore cannot establish a TNF-α-driven mechanism. Moreover, evidence supporting infliximab for ICI-associated myocarditis remains limited, and TNF-α inhibition raises cardiovascular safety concerns, particularly in patients with heart failure (35–37). Clinical recovery occurred after multiple sequential and overlapping interventions; therefore, the contribution of any individual treatment, including infliximab, cannot be determined.

The failure of cTnT to completely normalize presented a significant management challenge during the tapering phase. Although cTnT levels decreased substantially during treatment, they remained above the upper reference limit. Following discontinuation of corticosteroids and mycophenolate mofetil, the patient developed recurrent chest discomfort accompanied by an increase in cTnT to 299.4 ng/L. Restarting corticosteroid-based immunosuppression — prompted by both the symptoms and the biomarker trend — brought cTnT back down. Serial troponin measurement is a standard tool for tracking ICI-associated myocardial injury (38–44), and biomarker rebound after treatment reduction has been described in other patients with persistent immune-mediated cardiac injury (45–47). Still, cTnT must be read cautiously here: skeletal-muscle involvement was clinically suspected, and cardiac troponin I was not available for comparison. This recurrence highlights the uncertainty surrounding the optimal duration and tapering of immunosuppressive treatment in patients with persistent biomarker abnormalities. Tapering decisions in such patients likely need to weigh symptoms, ECG findings, ventricular function, biomarker trends, neuromuscular recovery, and treatment toxicity together, rather than resting on any single threshold.

Two episodes of pneumonia occurred during prolonged corticosteroid and mycophenolate therapy despite TMP-SMX prophylaxis against *Pneumocystis jirovecii*: the first caused by Klebsiella pneumoniae and the second by *K. pneumoniae* with *Candida albicans*.

Opportunistic and bacterial infections are recognized complications of prolonged glucocorticoid and additional immunosuppressive therapy (48, 49), underscoring the need to balance control of immune-mediated myocardial injury against infectious toxicity. The 2022 ESC cardio-oncology guidelines recommend baseline ECG and cardiac troponin assessment before ICI therapy, with baseline echocardiography particularly indicated in high-risk patients. Serial ECG and troponin measurements should be considered before ICI doses 2, 3, and 4 and, if normal, every three doses thereafter. New cardiovascular or neuromuscular symptoms, ECG abnormalities, or biomarker elevation should prompt urgent multidisciplinary assessment, including echocardiography and CMR when feasible, together with targeted evaluation for overlapping neuromuscular toxicity (33, 50, 51). Sintilimab was permanently discontinued because of the life-threatening cardiovascular toxicity (52, 53). This report has limitations. First, myocarditis was diagnosed clinically, without histological or CMR confirmation. Second, neuromuscular investigations were incomplete because of clinical deterioration and the patient's reluctance to undergo additional testing, and acetylcholine receptor antibody testing was unavailable at our institution (26, 54). Cardiac troponin I was not measured, and the absence of a baseline echocardiogram leaves open whether radiotherapy or paclitaxel had already left some subclinical imprint on this heart before ICI exposure. Because plasma exchange was limited in volume and given alongside several other immunosuppressive agents, we cannot attribute the patient's recovery to any single treatment. Whether the persistent mild cTnT elevation reflects ongoing low-grade myocardial inflammation, early fibrosis, or a new stable baseline remains uncertain and requires longer follow-up. Neither acute-phase nor follow-up CMR was obtained because of initial clinical instability and subsequent patient reluctance to undergo additional investigations after substantial treatment-related expenses, limiting assessment of myocardial inflammation and residual fibrosis.

## 4 Conclusion

Sintilimab-associated myocarditis may present with rapid progression of conduction-system disease, including complete atrioventricular block and hemodynamic instability, even when left ventricular systolic function is relatively preserved. When neuromuscular symptoms coexist, clinically suspected overlap syndrome should prompt careful multidisciplinary evaluation while acknowledging diagnostic uncertainty when confirmatory testing is unavailable. Furthermore, managing severe ICI-associated myocarditis requires navigating the delicate balance between preventing recurrent myocardial injury during immunosuppressive tapering and minimizing the substantial risk of opportunistic infections associated with prolonged treatment. Close ECG surveillance, serial biomarker assessment, individualized immunosuppressive management, and long-term follow-up are therefore essential in severe ICI-associated myocarditis.

## 5 Patient perspective

The patient recalled experiencing profound fear and a sense of impending death during the initial episode of severe chest tightness and dyspnea. While he noted significant relief of fatigue and chest pain following the initiation of immunosuppressive therapy, the recurrence of symptoms during the medication taper caused considerable anxiety regarding the potential for irreversible heart failure and the ensuing financial burden of prolonged care.

## Data Availability

The original contributions presented in the study are included in the article/Supplementary Material, further inquiries can be directed to the corresponding authors.

## Supplementary material

FIGURE S1. INITIAL DIAGNOSTIC IMAGING FINDINGS OF THE PATIENT. (A) Contrast-enhanced chest CT obtained on December 24, 2024, showing a 25 mm × 19 mm nodular lesion in the right pulmonary hilum. (B) PET/CT scan obtained on December 20, 2024, revealing a 29 mm × 23 mm nodule with an SUVmax of 19.5 near the right middle lobe hilum.
